# An Indoor Robust Localization Algorithm Based on Data Association Technique

**DOI:** 10.3390/s20226598

**Published:** 2020-11-18

**Authors:** Long Cheng, Yong Wang, Mingkun Xue, Yangyang Bi

**Affiliations:** 1 Department of Computer and Communication Engineering, Northeastern University, Qinhuangdao 066004, China; wangyong232513@163.com (Y.W.); mingkunxue@gmail.com (M.X.); 2 SANY Group CO., Ltd., Changping, Beijing 102202, China; biyy@sany.com.cn

**Keywords:** non-line-of-sight, data association technique, localization, robustness

## Abstract

As a key technology of the Internet of Things, wireless sensor network (WSN) has been used widely in indoor localization systems. However, when the sensor is transmitting signals, it is affected by the non-line-of-sight (NLOS) transmission, and the accuracy of the positioning result is decreased. Therefore, solving the problem of NLOS positioning has become a major focus for indoor positioning. This paper focuses on solving the problem of NLOS transmission that reduces positioning accuracy in indoor positioning. We divided the anchor nodes into several groups and obtained the position information of the target node for each group through the maximum likelihood estimation (MLE). By identifying the NLOS method, a part of the position estimates polluted by NLOS transmission was discarded. For the position estimates that passed the hypothesis testing, a corresponding poly-probability matrix was established, and the probability of each position estimate from line-of-sight (LOS) and NLOS was calculated. The position of the target was obtained by combining the probability with the position estimate. In addition, we also considered the case where there was no continuous position estimation through hypothesis testing and through the NLOS tracking method to avoid positioning errors. Simulation and experimental results show that the algorithm proposed has higher positioning accuracy and higher robustness than other algorithms.

## 1. Introduction

In recent years, wireless sensor network (WSN) has been applied to target tracking, navigation, industrial monitoring, emergency services, and other fields due to the simple deployment of nodes, complete functions, and flexible network settings. With the rapid development of embedded systems, it is being applied to a wider range of applications. WSN positioning refers to using pre-deployed sensor nodes (anchor nodes) to obtain data, obtaining the coordinate information of the positioning target through a positioning algorithm. The positioning methods are roughly divided into two categories: ranging and no ranging. The ranging methods include time of arrival (TOA) [1], time difference of arrival (TDOA) [2], received signal strengthen indicate (RSSI) [3], angle of arrival (AOA) [4], and so on. No ranging methods include centroid algorithm, distance vector (DV)-hop [5] algorithm, and so on. The distance information obtained by the sensor is used as the input of the positioning algorithm, and the position of the target is obtained through the algorithm processing to complete the positioning work. In the process of signal propagation, due to various obstacles in the propagation environment, the signal is affected by non-line-of-sight (NLOS) transmission. NLOS transmission refers to when the transmission signal encounters an obstacle during transmission, and the signal reaches the receiving end through reflection, refraction, and diffraction. The distance measured by the sensor is greater than the actual distance. NLOS transmission can reduce positioning accuracy, especially in the indoor positioning, and this effect is particularly serious. Therefore, how to reduce the impact of NLOS on positioning results and obtain higher positioning accuracy is the main research hot spot of indoor positioning algorithms.

The proposed algorithm in this paper aims to solve the effect of NLOS transmission that reduces the accuracy of indoor localization. We used the concept of grouping by dividing the anchor nodes involved in positioning into several subgroups, screened out the position estimation that met the conditions by using the method of hypothesis testing, and then calculated the association probability through the improved data association method and obtained the position estimation of the target with the previous position estimation. In terms of the situation where the number of positions passing the hypothesis testing was zero continuously, an NLOS tracking method was proposed to avoid positioning errors. The advantages of the proposed algorithm are as follows:

- NLOS identification was performed through hypothesis testing, and some NLOS transmissions were eliminated, effectively reducing the effect of NLOS.
- The algorithm established the corresponding clustering probability matrix for the position estimation through hypothesis testing with the improved method to calculate the probability, where the positioning accuracy was higher.
- If the hypothesis testing failed continuously when there was no position estimation obtained from the subgroup that fell into the verification gate, the NLOS tracking method was used to avoid positioning errors.

The paper is organized as follows: Section 2 introduces related work, Section 3 describes the establishment of the signal model, Section 4 analyzes the proposed algorithm, and Section 5 discusses the simulation results.

## 2. Related Works

The indoor positioning problem has achieved fruitful results since the research began; scholars have proposed many newer algorithms in terms of NLOS recognition and weakening in recent years.

In terms of identifying NLOS, there are many methods. The NLOS signal reaches the receiving end through reflection or diffraction, thus the amplitude of range is longer than the LOS signal usually. In [6], the authors propose an algorithm that classifies and discards NLOS signals for indoor positioning and measurement systems. First, NLOS signals are identified based on the signal amplitude to obtain an initial probability estimate. Then, in the weight update step, iterative re-weighting of the least square regression is used to determine the receiving node position with the measured distance information. The author in [7] identifies the NLOS propagation through distance and angular probability, and a distance and angle probability model is proposed based on the sampling information to identify the NLOS propagation, followed by the maximum likelihood estimation (MLE) method to reduce the distance error in NLOS propagation through the model established previously. The improved Monte Carlo method is used to compute the best location of the mobile node, which reduces the computational complexity. Similar to [6], due to the strengths of the received signals being different between LOS and NLOS, the author in [8] derives an error model by performing a deviation and Cramer–Rao bound (CRB) analysis on the proposed LOS and positioning methods based on conventional radio signal strength. The proposed LOS-based method improves positioning accuracy by eliminating received signals that do not meet the required power. Furthermore, by estimating the LOS component through the fading signal, NLOS interference to the positioning result can be suppressed. Using data analysis to obtain some character of NLOS propagation to identify the NLOS propagation in [9], the author uses machine learning (ML) technology to analyze several sets of actual ultra-wide band (UWB) measurements captured in different situations in an attempt to identify measurements of NLOS propagation conditions. Therefore, the deviation between the measured value and the actual value is reduced. The results show that when LOS exists between the transmitter and the receiver, the ML technology is not only suitable for identifying the NLOS propagation conditions but also can alleviate the estimation error. Ranging residual is always used to identify NLOS. While in [10], the author compares the ranging residual compensation to some NLOS error mutations to identify LOS and NLOS, Kalman filter (KF) is performed within the distance from the LOS environment, and in the NLOS environment, a biased KF based on ranging residual compensation is used. The author in [11] proposes a distributed residual weighted discrimination algorithm. Position estimation for groups containing NLOS propagation errors are always distributed in isolation, and estimations without NLOS propagation errors are mostly concentrated in a small range. Based on this feature, the author proposes a two-step least squares method to determine the optimal location through analyzing the distribution of estimated coordinates.

Combination algorithms that weaken NLOS transmission are becoming a research hotspot. First, getting an estimation of the target node through different filters, which is handled by a different algorithm, is an effective choice. In [12], the authors propose an indoor positioning method based on a track quality-based fusion algorithm. First, extended Kalman filter (EKF) and robust extend Kalman filter (REKF) are used to obtain the position estimation of the mobile node followed by two KFs to further filter the estimates, which is the input of the fusion algorithm to obtain the final position estimation of the mobile target. Similar to [12], the authors propose a hyperbolic weighted centroid algorithm which is based on the TDOA localization model in [13]. The measured value of the TDOA distance difference is filtered by a KF firstly and then through the hyperbolic positioning algorithm to obtain an initial solution. In order to further reduce the influence of NLOS, an effective weighted centroid technique is used to calculate the final target position. In the positioning algorithm, the outlier is a pivotal factor which greatly affects the accuracy of algorithm. The authors in [14] combine EKF and outlier detection for sensor fusion technology to avoid the problem, while the authors in [15] solve this problem by iterative least squares algorithm. The authors in [15] obtain relatively accurate initial values firstly through the genetic algorithm, then, the iterative least square algorithm is used to update the position estimate so that the estimated value converges to the expected value and further improves the positioning accuracy. The authors in [16] propose a novel method that uses a combination of measurements of RSSI and the measurement of AOA two factors to resolve the problem of target localization. If the transmit power is known, by using the polarization identity, the AOA measurements collected are converted into the norm form, and a new relationship is established based on the AOA measurements and the unknown target position by using a second-order method to obtain the location of the target.

Improving the shortcomings of the exiting algorithm is also becoming a popular research topic. In [17], the authors transform the new robust weighted least squares problem into a non-convex optimization problem through the S-Lemma, so that the optimal target position is obtained. Because the frequency of signal is hard to affect during propagation, the authors in [18] establish a frequency and position transfer function by linking the field where a receiver is given to the source, which not only mitigates the NLOS effect but also calibrates the propagation channel back to free space, thus the performance is better than the usual TDOA positioning algorithm. For the purpose of avoiding the shortcomings of the robust least square (RLS) methods and reducing the upper limit of the NLOS error, the source location and the NLOS error in the predicted path are jointly estimated in [19]. To avoid the use of triangular inequality by the S-Lemma, the final target position could be obtained through the optimization problems. In [20], in order to deal with the abnormal row and column structure in the multi-dimensional similarity (MDS) matrix caused by NLOS propagation, the authors propose an improved robust matrix approximation program that uses the 2,1-norm and applies alternating directions of multipliers method to resolve the resulting nonlinear constraint optimization problem. Voting is a good idea that can be used to mitigate the influence of NLOS. In [21,22,23], the author proposes layered voting ideas to perform state detection and distance correction. This method can decrease the impact of NLOS propagation well and employs different filters to filter the values obtained in the previous step by the method of MLE to get the position estimate of the mobile node.

## 3. Signal Model

Suppose that the mobile node moves in a limited two-dimensional plane, and *M* anchor nodes are deployed randomly. $\hat{x}\left(k\right)={\left[\begin{array}{cccc} x\left(k\right) & y\left(k\right) & \tilde{x}\left(k\right) & \tilde{y}\left(k\right) \end{array}\right]}^{T}$ is the state vector of the mobile node, and (x(k),y(k)) is the coordinate of the mobile node at *k*-th time. $\left(\tilde{x}\left(k\right),\tilde{y}\left(k\right)\right)$ represents the velocity along the x-axis and the y-axis at *k*-th time. Therefore, the motion change of the target node can be described by the change of state vector. The change of the state vector of the target node is modeled as:

(1) $$\hat{x}\left(k\right)=G\hat{x}\left(k-1\right)+C\varpi \left(k-1\right)\;G=\left[\begin{array}{cc} \begin{array}{cc} \begin{array}{c} 1\\ 0 \end{array} & \begin{array}{c} 0\\ 1 \end{array} \end{array} & \begin{array}{cc} \begin{array}{c} \Delta t\\ 0 \end{array} & \begin{array}{c} 0\\ \Delta t \end{array} \end{array}\\ \begin{array}{cc} \begin{array}{c} 0\\ 0 \end{array} & \begin{array}{c} 0\\ 0 \end{array} \end{array} & \begin{array}{cc} \begin{array}{c} 1\\ 0 \end{array} & \begin{array}{c} 0\\ 1 \end{array} \end{array} \end{array}\right]C=\left[\begin{array}{cc} \begin{array}{c} \Delta t^{2}/2\\ 0\\ \Delta t\\ 0 \end{array} & \begin{array}{c} 0\\ \Delta t^{2}/2\\ 0\\ \Delta t \end{array} \end{array}\right]$$

where Δt represents the sampling interval, and $I_{M}$ is the M×M identity matrix, and the driving noise ϖ(k) is modeled as Gaussian white noise with the mean of 0 and a covariance matrix of $Q\left(k\right)=\sigma_{\omega }^{2}I_{2}$, where k=1,2,…,K describes the uncertainty on the motion model at time k. G represents the state transition matrix of the mobile node, and C represents the interference noise input matrix. The distance measurement value between the *M* anchor nodes and the mobile node at the moment k is expressed as: $D\left(k\right)={\left[d_{1}\left(k\right),\ldots ,d_{M}\left(k\right)\right]}^{T}$ if the probability that the NLOS measurement occurs is α, the probability that the LOS measurement appears as 1−α, $d_{L}\left(k\right)$ represents the distance measured from the *L*-th anchor node to the mobile node, recorded as [24]:

(2) $$d_{L}\left(k\right)=\{\begin{array}{c} h_{L}\left(\hat{x}\left(k\right)\right)+v\left(k\right)\;\;\;\;\;\;\;\;\;\;\;\;\;if\;LOS\;condition\\ h_{L}\left(\hat{x}\left(k\right)\right)+n_{NLOS}+v(k)\;\;\;\;\;if\;NLOS\;condition \end{array}$$

$h_{L}(\hat{x}(k))$ represents the actual geometric distance from the *L*-th anchor node which participates in positioning to the target node, which is defined as:

(3) $$h_{L}\left(\hat{x}\left(k\right)\right)=\sqrt{{\left(x\left(k\right)-x_{L}\right)}^{2}+{\left(y\left(k\right)-y_{L}\right)}^{2}}\;L=1\;,\;2\;,\ldots ,\;M$$

where $\left(x_{L},y_{L}\right)$ represents the two-dimensional coordinates of the *L*-th anchor node, $n_{NLOS}$ represents the NLOS deviation, and v(k) is the LOS noise modeled as Gaussian white noise with mean of 0 and standard deviation of $\sigma_{G}^{2}$. The Euclidean distance set from the mobile node to the M anchor nodes is $h\left(\hat{x}\left(k\right)\right)={\left[h_{1}\left(k\right),\ldots ,h_{M}\left(k\right)\right]}^{T}$.

## 4. Proposed Algorithm

### 4.1. General Concept

**Figure 1:**
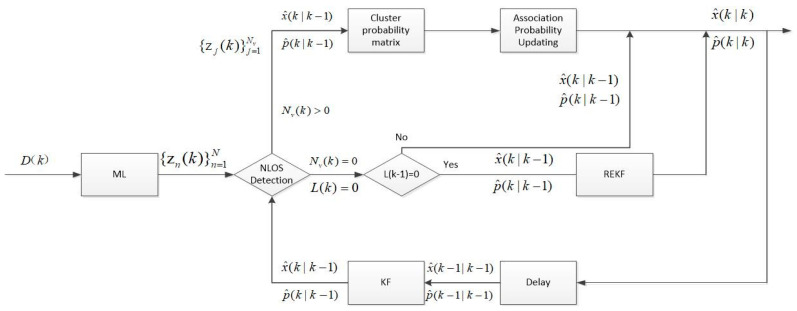
The flow chart of the algorithm.

The flow chart of the proposed algorithm is shown in Figure 1. In this paper, we divided the M anchor nodes into $N=C_{M}^{3}$ different subgroups and through the MLE method to obtain the position estimation of the target node $z_{n}\left(k\right)={\left[x_{n}\left(k\right),y_{n}\left(k\right)\right]}^{T}$ in each subgroup. Because the propagation environment from the anchor node to the target node in the subgroup n was unknown, if it was NLOS, the position estimation accuracy of the target node calculated by the corresponding position estimation was decreased, and the position estimation in the LOS state had higher accuracy. Therefore, we performed hypothesis testing on the position estimates obtained for each subgroup and reduced the effect of NLOS errors through hypothesis testing in advance. When performing hypothesis testing at time k, Kalman prediction state vector $\hat{x}(k|k-1)$ and covariance matrix P(k|k−1) were needed to obtain the predicted position estimate z(k|k−1) of the target node. A statistical test $T_{i}\left(k\right)$ was calculated using the obtained predicted position estimate z(k|k−1), and the value was compared with a preset threshold. If the statistical test value was less than the threshold, the assumption was true, and the position estimate was used in subsequent positioning calculations. If the statistical test value was larger than the threshold set previously, the assumption was not true, and the corresponding position estimation was discarded. The selection of the threshold was decided on the threshold probability $P_{G}$. The number of statistically estimated position estimates through hypothesis testing was $N_{V}\left(k\right)$. If $N_{V}\left(k\right)$ was greater than 0, a corresponding clustering probability matrix was established, the associated probability was calculated using an improved method, and the state of the target node was updated using the associated probability and the position estimation through hypothesis testing. If $N_{V}\left(k\right)$ equaled 0, it meant that the position estimates had not passed the hypothesis testing, and then it was detected whether the number of position estimates that passed the hypothesis testing at the previous moment was 0 or not. If it was 0, the position estimate of the mobile node was calculated by the NLOS tracking method; otherwise, the predicted position estimate was used as the position estimation of the mobile node. In this way, it was possible to avoid the situation of continuous positioning errors caused by hypothesis testing without position estimation and strengthen the robustness. The components of the algorithm are described in detail below.

### 4.2. Grouping and Hypothesis Testing

This article assumed that there were M anchor nodes participating in the positioning of the target node, and that M was greater than 3. Anchor nodes were grouped into groups of three, with a total of N subgroups. When sampling was performed at k moment, the distance measurement from the anchor node to the target node was also divided into N subgroups. The MLE was performed on the distance measurement obtained by each subgroup to get the position estimate of the target node. The specific process of hypothesis testing is described in detail below.

#### 4.2.1. Kalman Prediction

We used the state vector $\hat{x}\left(k-1|k-1\right)$ and the covariance matrix P(k−1|k−1) of the mobile node at time k−1 to predict the state vector and the covariance matrix of the mobile node at time k through the next formula:

(4) $$\hat{x}\left(k|k-1\right)=G\hat{x}\left(k-1|k-1\right)$$

(5) $$P\left(k|k-1\right)=GP\left(k-1|k-1\right)G^{T}+CQC^{T}$$

The predicted position estimation of the mobile node is:

(6) $$z\left(k|k-1\right)=B\hat{x}\left(k|k-1\right)\;B=\left[\begin{array}{cccc} 1 & 0 & 0 & 0\\ 0 & 1 & 0 & 0 \end{array}\right]$$

B is the observation matrix. The difference between the estimated position of the target node and the position estimation of the target node obtained by the nth subgroup is defined as innovation:

(7) $$v_{n}\left(k\right)=z_{n}\left(k\right)-z\left(k|k-1\right)\;n=1\;,\;2\;,\ldots ,\;M$$

#### 4.2.2. NLOS Identification

At time k, if the anchor nodes in the n-th subgroup are all in LOS environment, the innovation meets:

(8) $$v_{n}\left(k\right)\sim N\left(0,S_{n}\left(k\right)\right)\;n=1\;,\ldots ,\;N$$

The covariance of the innovation is:

(9) $$S_{n}\left(k\right)=BP\left(k|k-1\right)B^{T}+\sigma_{G}^{2}I_{2}*{\left(H_{n}^{T}\left(k\right)H_{n}\left(k\right)\right)}^{-1}\;H_{n}\left(k\right)=\frac{\partial h_{n}\left(\hat{x}\left(k\right)\right)}{\partial \hat{x}\left(k\right)}$$

In order to identify (8), we defined the following assumptions:

(10) $$H_{0,n}:\nu_{n}(k)\sim N(0,S_{n}(k))\;n=1\;,\ldots ,\;N$$

(11) $$H_{1,n}:nonH_{0,n}n=1\;,\ldots ,\;N$$

If the anchor nodes in the n-th subgroup are all in the LOS environment, the assumption $H_{0,n}$ is true. If at least one anchor node in the n-th subgroup is in the NLOS environment, the $H_{1,n}$ is true, and the position estimate obtained by the subgroup will have a large error. If the anchor nodes in the n-th subgroup are in the LOS environment, the obtained position estimation will fall into the verification gate. In order to identify the position estimates obtained for each subgroup, we defined a statistical test as:

(12) $$T_{n}(k)=v_{n}^{T}\left(k\right)S_{n}^{-1}\left(k\right)v_{n}\left(k\right)\;n=1\;,\ldots ,\;N$$

We compared the statistical test value with a preset threshold value. If the statistical test value was larger than the threshold, the corresponding hypothesis was invalid, otherwise, it was true. The selection of the threshold of the correlation gate is related to the threshold probability $P_{G}$:

(13) $$\int_{0}^{\gamma }f_{\chi^{2}\left(2\right)}\left(x\right)dx=P_{G}=1-P_{FA}$$

Threshold probability $P_{G}$ is the probability that the position estimate from the LOS environment falls into the verification gate. $f_{\chi^{2}\left(2\right)}\left(\cdot \right)$ is a chi-square distribution with a degree of freedom of 2, and $P_{FA}$ is the alert probability when the probability that the position estimation falls into the verification door is estimated to be an LOS environment no less than 0.99, and the threshold of the chi-square distribution with a degree of freedom of 2 is γ=9.21. x represents the location estimation to be tested. We then calculated the number of position statistics that passed the hypothesis testing and recorded as $N_{v}\left(k\right)$. If it was greater than 0, the subsequent calculation of the correlation probability was performed in addition to updating the state estimation and the covariance matrix. If $N_{v}\left(k\right)$ was 0, and $N_{v}\left(k-1\right)$ was 0, at this time, the final position estimate was obtained by the NLOS tracking method. Otherwise, the predicted position estimate was used as the final position estimate.

### 4.3. Association Probability Updating

This article used the improved probability data association method to calculate the association probability. First, the correlation event was defined:

- The LOS environment and the NLOS environment from the target node to the anchor node had position estimates;
- Each position estimation was from LOS or NLOS environments.

Defining the associated event:

$\xi_{lt}:$ {Hypothesis-tested position estimates from LOS environment}, $l=1\;,\ldots ,\;N_{V}(k)$, t=1. The event $\xi_{01}$ indicates that the position estimation from the LOS environment does not fall into the verification gate, $\xi_{l0}$ indicates that the position estimation $z_{l}\left(k\right)$ is from a NLOS environment, and $\xi_{00}$ indicates that there is no position estimation in the NLOS environment that is meaningless. The correct position at time k is estimated as: $Z\left(k\right)={\left\{z_{l}\left(k\right)\right\}}_{l=1}^{N_{v}\left(k\right)}$, $Z^{k}=\left\{Z\left(1\right),Z\left(2\right),\ldots ,Z\left(k\right)\right\}$ represents correct positions from start time to current time. The probability density function of the event $\left(l=1,\ldots ,N_{v}\left(k\right),t=1\right)$ is modeled as:

(14) $$f_{l1}\left(z_{l}\left(k\right)|\xi_{l1}\left(k\right),N_{v}\left(k\right),Z^{k}\right)==P_{G}^{-1}N\left(z_{l}\left(k\right);z\left(k|k-1\right),S_{l}\left(k\right)\right)==P_{G}^{-1}\frac{\exp\left\{-\frac{1}{2}v_{l}^{T}\left(k\right)S_{l}^{-1}\left(k\right)v_{l}\left(k\right)\right\}}{2\pi {\left|S_{l}\left(k\right)\right|}^{0.5}}$$

The probability density function of the event $\xi_{l0}\left(l=1\;,\ldots ,\;N_{v}\left(k\right)\right)$ is assumed as:

(15) $$f_{l0}\left(z_{l}\left(k\right)|\xi_{l0}\left(k\right),L\left(k\right),Z^{k}\right)=\frac{N_{v}\left(k\right)}{V\left(k\right)}\;l=1\;,\ldots ,\;N_{v}\left(k\right)$$

(16) $$V\left(k\right)=\pi \tau {\left|S\left(k\right)\right|}^{0.5}$$

V(k) is the acreage of the verification area corresponding to the accepted hypothesis. The probability density function of the event $\xi_{01}$ is:

(17) $$f_{01}\left(z_{l}\left(k\right)|\xi_{01}\left(k\right),N_{v}\left(k\right),Z^{k}\right)={\left(2V\right)}^{-1}\left(1-P_{d}P_{G}\right)$$

where $P_{d}$ is the detected probability, which represents the probability that the position estimation falls into the verification area which can be detected by the verification gate.

The probability density function of the event $\xi_{00}$ is:

(18) $$f_{00}\left(z_{l}\left(k\right)|\xi_{00}\left(k\right),N_{v}\left(k\right),Z^{k}\right)=0$$

Association probability is modeled as:

(19) $$\beta_{l1}\left(k\right)=\frac{1}{c}\left[\varepsilon_{l1}\left(k\right)\cdot \prod_{\begin{array}{c} tr=0\\ tr\neq 1 \end{array}}^{1}\sum_{\begin{array}{c} r=0\\ r\neq l \end{array}}^{N_{v}\left(k\right)}\varepsilon_{rtr}\left(k\right)+\zeta_{l1}\left(k\right)\prod_{\begin{array}{c} r=0\\ r\neq l \end{array}}^{N_{v}\left(k\right)}\sum_{\begin{array}{c} tr=0\\ tr\neq 1 \end{array}}^{1}\zeta_{rtr}\left(k\right)\right]$$

where tr=0,1,r=0,1,…L(k)

(20) $$\varepsilon_{lt}\left(k\right)=f_{lt}\left(z_{l}\left(k\right)|\xi_{lt}\left(k\right),N_{v}\left(k\right),Z^{k}\right)/\sum_{l=0}^{N_{v}\left(k\right)}f_{lt}\left(z_{l}\left(k\right)|\xi_{lt}\left(k\right),N_{V}\left(k\right),Z^{k}\right)$$

(21) $$\zeta_{lt}\left(k\right)=f_{lt}\left(z_{l}\left(k\right)|\xi_{lt}\left(k\right),N_{v}\left(k\right),Z^{k}\right)/\sum_{t=0}^{1}f_{lt}\left(z_{l}\left(k\right)|\xi_{lt}\left(k\right),N_{v}\left(k\right),Z^{k}\right)$$

where *c* is the normalization factor. Kalman gain K(k) needs to be calculated during the state updating:

(22) $$K\left(k\right)=P\left(k|k-1\right)B^{T}S^{-1}\left(k\right)$$

and the updated state estimate of the mobile node and the updated covariance matrix are obtained by the associated probability:

(23) $$\hat{x}\left(k|k\right)=\hat{x}\left(k|k-1\right)+K\left(k\right)\sum_{l=1}^{L\left(k\right)}\beta_{l1}\left(k\right)v_{l}\left(k\right)$$

(24) $$P\left(k|k\right)=\beta_{01}\left(k\right)P\left(k|k-1\right)+\left(1-\beta_{01}\left(k\right)\right)P_{c}\left(k\right)+\tilde{P}\left(k\right)$$

(25) $$P_{c}\left(k\right)=\left(I_{4}-K\left(k\right)B\right)P\left(k|k-1\right)$$

(26) $$\tilde{P}\left(k\right)=K\left(k\right)\left[\sum_{l=1}^{N_{v}\left(k\right)}\beta_{l1}\left(k\right)v_{l}\left(k\right)v_{l}^{T}\left(k\right)-v\left(k\right)v^{T}\left(k\right)\right]K^{T}\left(k\right)$$

(27) $$v\left(k\right)=\sum_{l=1}^{N_{v}\left(k\right)}\beta_{l1}\left(k\right)v_{l}\left(k\right)$$

### 4.4. NLOS Tracking

When the hypothesis test occurred and there was no position estimation passing the hypothesis testing continuously, if the final position estimation was also calculated by the above method at this time, it caused a positioning failure. Therefore, if such a situation occurred, the final position estimation needed to be corrected by the NLOS tracking method. After each hypothesis testing was completed, a corresponding flag was set. If the number of position estimates passed by the hypothesis testing was greater than 0, the corresponding flag was recorded as 1. If it was 0, the corresponding flag bit was recorded as 0. If two 0′s appeared consecutively, REKF was used to calculate the target’s position estimate. We wrote the EKF into linear regression and rewrote Equations (1) and (2) as:

(28) $$\left[\begin{array}{c} I_{4}\\ H(k) \end{array}\right]*\hat{x}(k)=\left[\begin{array}{c} G\hat{x}(k-1|k-1)\\ y(k)-h(\hat{x}(k|k-1))+H(k)\hat{x}(k|k-1) \end{array}\right]+e(k)$$

(29) $$e(k)=\left[\begin{array}{c} G(x(k-1)-\hat{x}(k-1|k-1))+C\varpi (k-1)\\ -v(k) \end{array}\right]$$

(30) $$H(k)=\frac{\partial h(x(k))}{\partial x(k)}$$

(31) $$E[e(k)e^{T}(k)]=\left[\begin{array}{cc} P(k|k-1) & 0\\ 0 & R(k) \end{array}\right]=C(k)C^{T}(k)$$

Utilizing the bridge Cholesky decomposition, C(k) can be solved, and the linear regression model can be written as:

(32) $$\tilde{y}=Dx+b\tilde{f}v+\tilde{v}$$

(33) $$\tilde{y}=C^{-1}(k)\left[\begin{array}{c} \hat{x}(k|k-1)\\ y(k)-h(\hat{x}(k|k-1))+H(k)\hat{x}(k|k-1) \end{array}\right]$$

(34) $$D=C^{-1}(k)\left[\begin{array}{c} I_{4}\\ H(k) \end{array}\right]$$

(35) x=x(k)

(36) $$\tilde{v}=-C^{-1}(k)e(k)$$

From the linear model, with the probability density function of $\tilde{v}$, the maximum likelihood estimation of x could be obtained by solving the following equations:

(37) $$\sum_{i=1}^{M+\dim(x)}{\left[D\right]}_{\mathrm{ij}}\times \varphi ({\tilde{y}}_{i}-\sum_{j^{\prime }=1}^{\dim(x)}{\left[D\right]}_{ij^{\prime }}x_{j^{\prime }})=0\;j=1,\ldots ,\dim(x)$$

where dim(x) means the dimension of $\hat{x}(k)$, $\varphi (\upsilon )=-\frac{{f^{\prime }}_{v}(\upsilon )}{f_{v}(\upsilon )}$. With the least square method to solve the above linear model, we obtained $\hat{x}(k|k)={\left(D^{T}D\right)}^{-1}D^{T}\tilde{y}$. In order to avoid the sensitivity of the least squares method to outliers and increase the robustness of the algorithm, the following methods were used to improve:

(1) Let l=0, getting the initial position estimate ${\hat{x}}_{0}$.
(2) Calculate the residual: $\hat{\tilde{v}}=\tilde{y}-D{\tilde{x}}_{l}$
(3) Use the $\hat{\tilde{v}}$ to estimate the range of ${\hat{\sigma }}_{\tilde{V}}$
(4) Update the μ and $\hat{x}$:
  $\mu =1/(1.25\max(|\psi^{\prime }(\hat{\tilde{v}}/{\hat{\sigma }}_{V})|))$
  ${\hat{x}}_{l+1}={\hat{x}}_{l}+\mu {\left(D^{T}D\right)}^{-1}D^{T}\psi (\hat{\tilde{v}}/{\hat{\sigma }}_{V})$
(5) Calculate $\left|\right|{\hat{x}}_{l+1}-{\hat{x}}_{l}||$, if the value is less than the ε set previously, the loop is stopped to obtain the final position estimation value. Otherwise, go to the second step and continue the cycle until it stops:
  where $\psi (\upsilon )=\{\begin{array}{c} \upsilon \\ b\tanh[0.5b(c_{2}-|\upsilon |)]\\ 0 \end{array}\mathrm{sgn}(\upsilon ),\begin{array}{c} |\upsilon |\leq c_{1}\\ c_{1}<|\upsilon |<c_{2}\\ |\upsilon |\geq c_{2} \end{array}$

## 5. Experiment Simulation

This section shows the simulation results of the algorithm we proposed. In the experiment, anchor nodes were randomly installed in the simulation region, and one target node moved along a fixed trajectory; the simulation scene was 100 m×100 m. The target node moved 100 steps at a time, the sampling interval was Δt=0.5 s, the initial state vector was $\hat{x}\left(0\right)={\left[\begin{array}{cccc} 1\;m & 19.99\;m & 1\;m/s & 0.5\;m/s \end{array}\right]}^{T}$, and the covariance matrix was $P\left(0\right)=I_{4}$. $I_{4}$ was the 4×4 identity matrix. The initial state vector of each subgroup was ${\hat{x}}_{n,j}\left(0\right)=\hat{x}\left(0\right)$, and the initial covariance matrix of the subgroup was $P_{n,j}\left(0\right)=P\left(0\right)$. The threshold probability was $P_{G}=0.99$, and the alert probability was $P_{FA}=0.01$, $P_{d}=0.95$. The probability of NLOS measurement occurred was $P_{NLOS}$. In this paper, robust interacting multiple model (RIMM) algorithm [25], REKF algorithm [26], and modified probabilistic data association (MPDA) algorithm [27] were used as comparison. In this paper, the root mean square error (RMSE) in (38) and the cumulative distribution function (CDF) were used as indicators to assess the algorithm.

(38) $$RMSE=\sqrt{\frac{1}{K}\cdot \frac{1}{MC}\sum_{j=1}^{MC}\sum_{k=1}^{K}\left({\left({\hat{x}}_{j}\left(k\right)-x_{j}\left(k\right)\right)}^{2}+{\left({\hat{y}}_{j}\left(k\right)-y_{j}\left(k\right)\right)}^{2}\right)}$$

$\left({\hat{x}}_{j}\left(k\right),{\hat{y}}_{j}\left(k\right)\right)$ was the position estimation obtained during the j-th operation, and $\left(x_{j}\left(k\right),y_{j}\left(k\right)\right)$ was the real position of the mobile node during the j-th operation. K=100 was the number of movements in each Monte Carlo simulation, and MC=1000 was the number of Monte Carlo runs. The simulation results of NLOS errors under different distributions are discussed below.

### 5.1. The NLOS Errors Obeys Gaussian Distribution

The measurement noise is modeled to be a Gaussian distribution $N\left(0,\sigma_{G}^{2}\right)$, and the NLOS error is a Gaussian distribution $N\left(\mu_{NLOS},\sigma_{NLOS}^{2}\right)$. The parameters of the Gaussian distribution are listed in Table 1.

**Figure 2:**
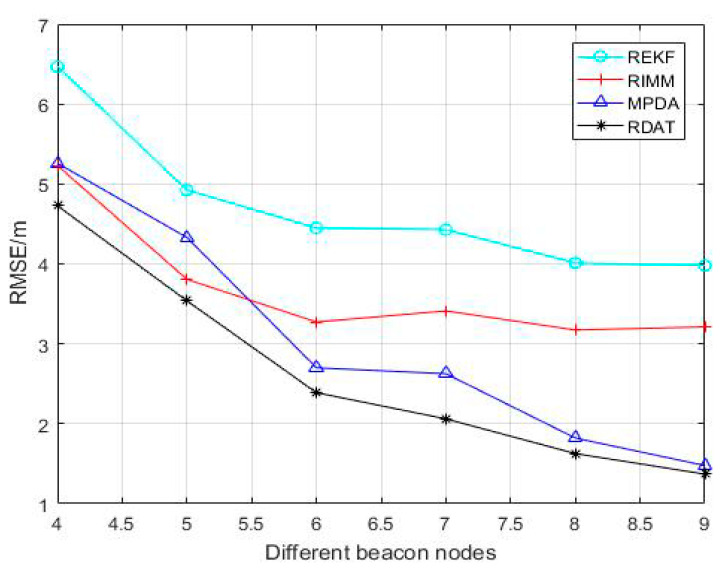
Performance comparison between the RIMM, robust extend Kalman filter (REKF), MPDA, and RDAT under different number of anchor nodes M, where $P_{NLOS}=0.5$,$N\left(0,1^{2}\right)$ and $N\left(5,6^{2}\right)$.

Figure 2 shows the change of the RMSE of the REKF algorithm, the RIMM algorithm, the MPDA algorithm, and the robust data association technique (RDAT) algorithm with the increase of the number of anchor nodes. The RMSEs of REKF, RIMM, MPDA, and RDAT all decreased as the number of anchor nodes increased. It can be concluded from the simulation diagram that the RMSEs of MPDA and RDAT dropped significantly. The average positioning errors of REKF, RIMM, MPDA, and RDAT were 4.710 m, 3.684 m, 3.036 m, and 2.619 m. The average positioning accuracies of RDAT were 44.39%, 28.91%, and 13.74% higher than those of REKF, RIMM, and MPDA, respectively.

**Figure 3:**
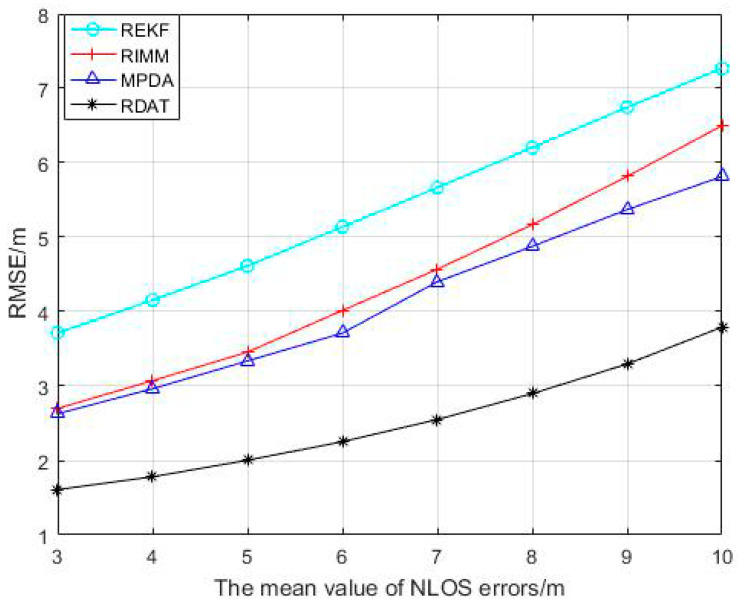
Simulation comparison between RIMM, REKF, MPDA, and RDAT with different probability of NLOS errors $P_{NLOS}$, $N\left(0,1^{2}\right)$ and $N\left(5,6^{2}\right)$.

Through Figure 3, the higher the probability of NLOS error that occurred, the larger the RMSE was, thus lowering the positioning accuracy. The average localization accuracies of REKF, RIMM, MPDA, and RDAT were 3.563 m, 2.565 m, 2.825 m, and 2.189 m, respectively. The positioning accuracies of RDAT were about 38.56%, 14.66%, and 22.51% higher than those of REKF, RIMM, and MPDA, respectively. Compared to the MPDA, due to the NLOS tracking method in the RDAT, the disadvantage of MPDA was alleviated.

**Figure 4:**
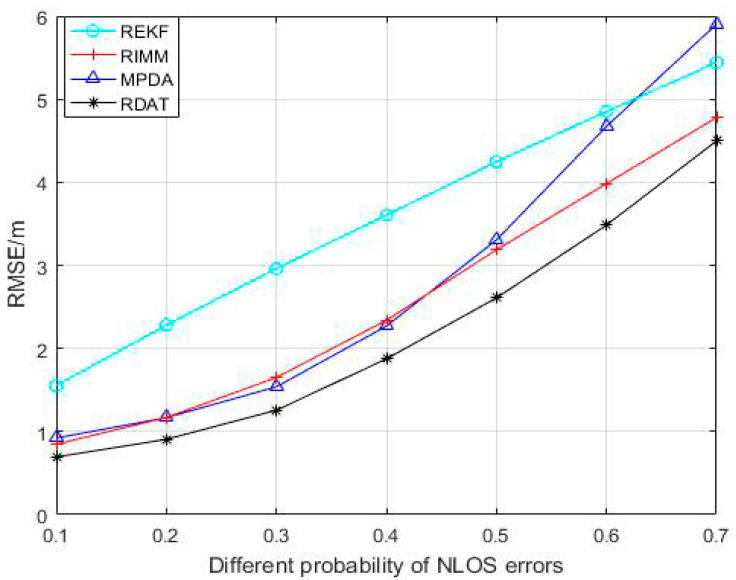
Performance comparison between RIMM, REKF, MPDA, and RDAT under different mean values of NLOS error $\mu_{NLOS}$, where $P_{NLOS}=0.5$, and $N\left(0,1^{2}\right)$, $N\left(5,6^{2}\right)$.

The mean value of the NLOS error changed from 3 to 10, and the RMSEs of REKF, RIMM, MPDA, and RDAT increased continuously in Figure 4, but RDAT increased more slowly than others with smaller positioning error. It can be concluded that the larger the average value of NLOS error was, the larger the error of positioning was; the performance of RDAT was still superior in the condition where the NLOS noise was higher.

**Figure 5:**
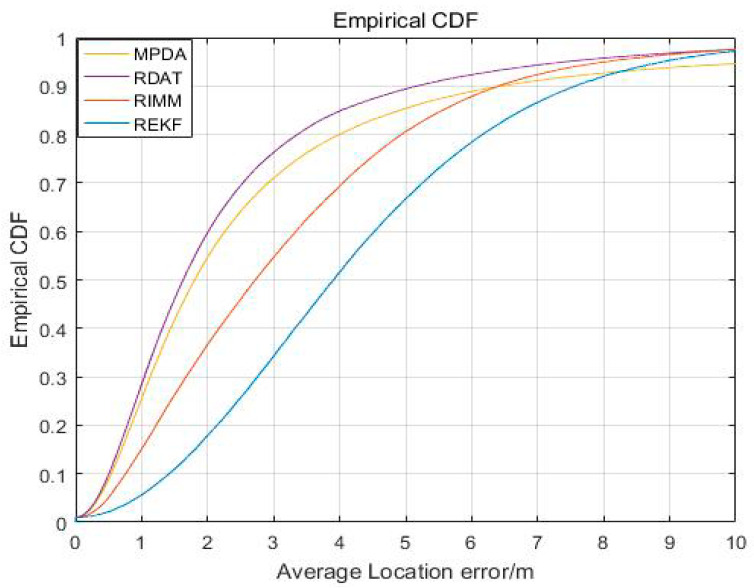
Performance contrast of RIMM, REKF, MPDA, and RDAT with different standard deviation of NLOS errors $\sigma_{NLOS}$, $P_{NLOS}=0.5$, $N\left(0,1^{2}\right)$ and $N\left(5,6^{2}\right)$.

It can be seen from the simulation in Figure 5 that when the standard deviation of NLOS error changed between 3 and 10, the RMSEs of the REKF and the RIMM algorithms rose steadily, while the RMSE of the MPDA algorithm decreased firstly and then increased. The RMSE of the RDAT algorithm was decreasing until the standard deviation reached nine, when it started to increase because the algorithm we proposed is sensitive to outliers. The average positioning accuracies of REKF, RIMM, MPDA, and RDAT were 4.398 m, 3.066 m, 3.466 m, and 2.075 m, respectively.

**Figure 6:**
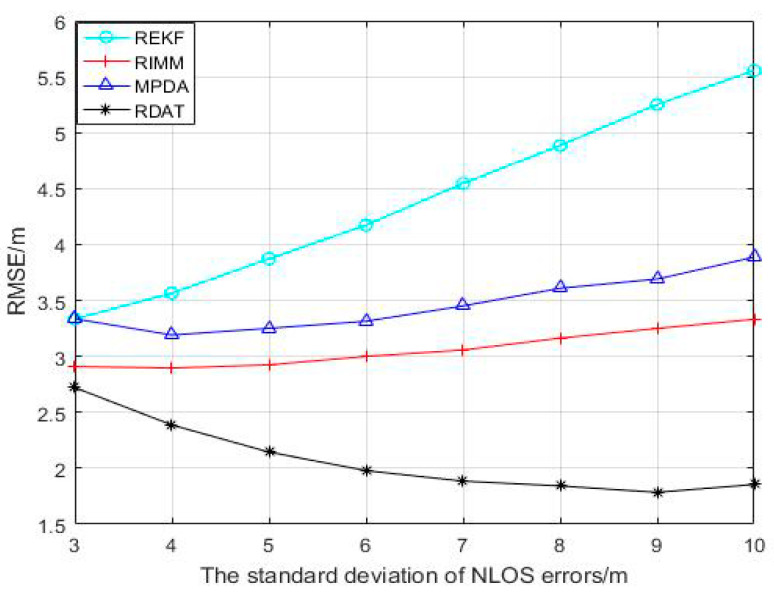
The cumulative distribution function (CDF) result of the localization error.

Figure 6 shows the cumulative error distribution of REKF, RIMM, MPDA, and RDAT. When the value of cumulative distribution function was 90%, the average localization accuracies of REKF, RIMM, MPDA, and RDAT did not exceed 7.569 m, 6.417 m, 6.463 m, and 5.168 m, respectively. This indicates the performance of RDAT was better than the others in most cases, as the accuracy of RDAT was significantly higher than REKF, RIMM, and MPDA.

### 5.2. The NLOS Errors Obey Uniform Distribution

Assuming that the measurement noise obeys the Gaussian distribution $N\left(0,\sigma_{G}^{2}\right)$, and the NLOS error is modeled as uniform distribution U(a,b), a and b are the minimum and the maximum of the uniform distribution, respectively. The default parameters of the uniform distribution are shown in Table 2.

**Figure 7:**
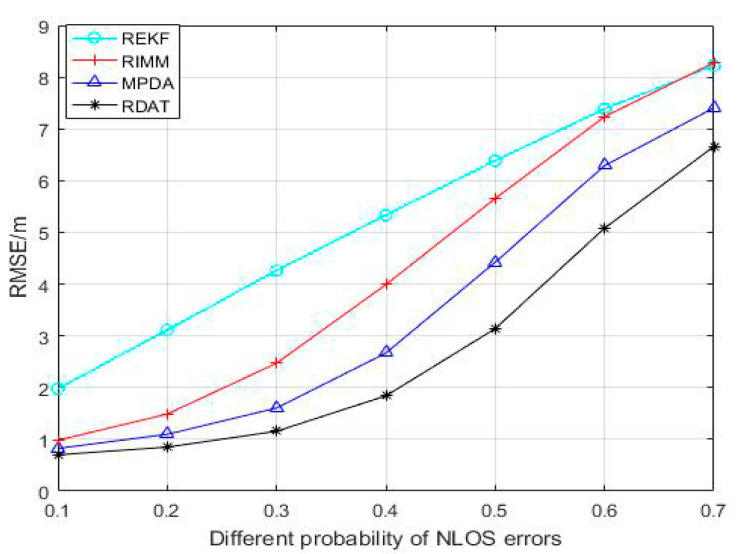
Performance comparison between RIMM, REKF, MPDA, and RDAT with the probability of NLOS errors $P_{NLOS}$, $N\left(0,1^{2}\right)$ and U(0,14).

With the occurrence probability of NLOS propagation changes between 0.1 and 0.7 in Figure 7, the position accuracy decreased while the RMSE increased. Compared with REFK and RIMM algorithms, MPDA and RDAT increased slowly; before 0.4, the change was small, and RDAT was better than MPDA. The average positioning accuracies of REKF, RIMM, MPDA, and RDAT were 5.241 m, 4.300 m, 3.473 m, and 2.775 m, respectively. The positioning accuracies of RDAT were about 47.05%, 35.47%, and 20.10% higher than REKF, RIMM, and MPDA, respectively.

**Figure 8:**
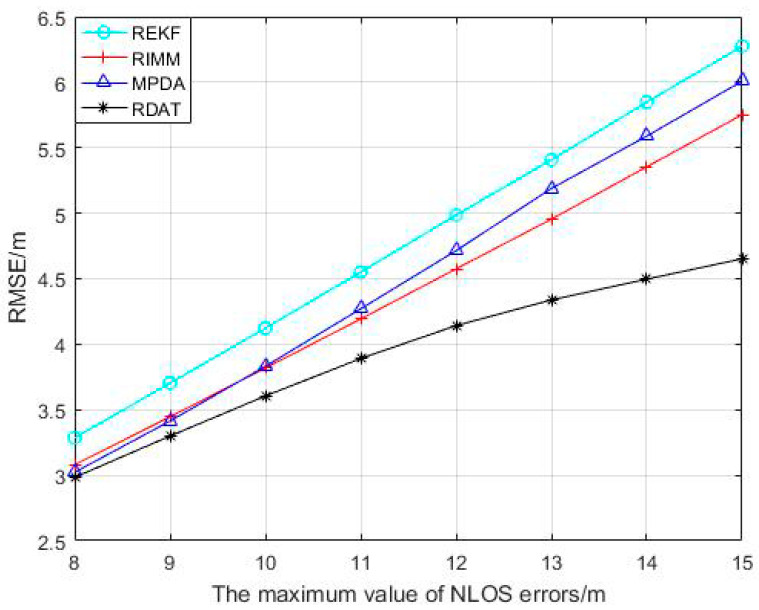
Performance comparison between RIMM, REKF, MPDA, and RDAT with different maximum value of NLOS error, where $P_{NLOS}=0.5$, $N\left(0,1^{2}\right)$ and U(0,14).

From Figure 8, with the increase of the maximum value of the NLOS error, compared to REKF, RIMM, and MPDA, the RDAT increased more slowly and did not change much. The average localization accuracies of REKF, RIMM, MPDA, and RDAT were 4.772 m, 4.397 m, 4.505 m, and 3.926 m, respectively.

### 5.3. The NLOS Errors Obey Index Distribution

The measurement noise is assumed to be a Gaussian distribution $N\left(0,\sigma_{G}^{2}\right)$, and the NLOS errors obey the index distribution E(λ) in this section. The default parameters of the index distribution are shown in Table 3.

**Figure 9:**
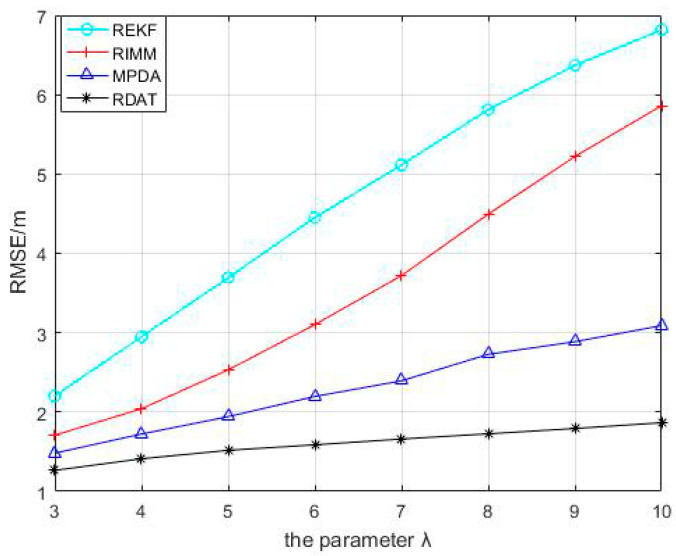
Performance contrast between RIMM, REKF, MPDA, and RDAT with different parameters of index distribution, where $P_{NLOS}=0.5$, $N\left(0,1^{2}\right)$ and E(8).

Referring to Figure 9, when the parameter of the index distribution increased from 3 to 10, REKF and RIMM increased faster, and the root mean square errors of MPDA and RDAT increased less. Compared with MPDA, RDAT was more stable. The average positioning errors of REKF, RIMM, MPDA, and RDAT were 4.676 m, 3.585 m, 2.305 m, and 1.603 m, respectively.

**Figure 10:**
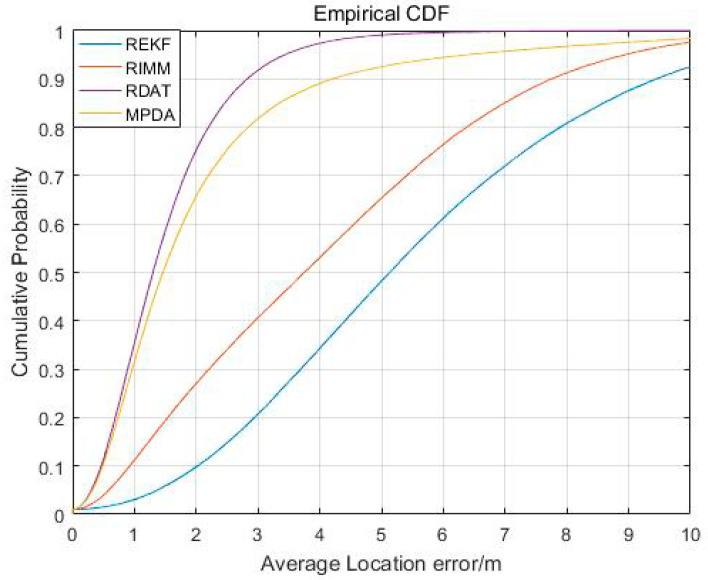
The CDF result of the localization error.

Figure 10 shows the average positioning error distribution of REKF, RIMM, MPDA, and RDAT when the NLOS errors followed an index distribution. The average positioning errors of 90% of REKF, RIMM, MPDA, and RDAT were not less than 9.459 m, 7.753 m, 4.208 m, and 2.833 m. When the cumulative distribution function approached one, the average positioning accuracy of RDAT did not exceed 6 m, which was a higher improvement than REKF, RIMM, and MPDA.

### 5.4. Experimental Result

**Figure 11:**
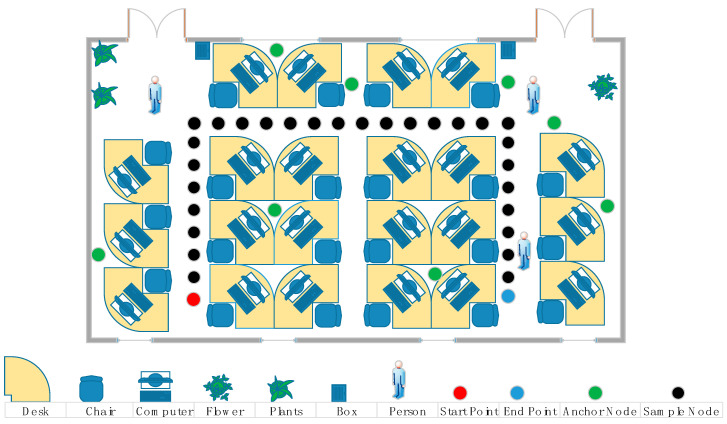
Deployment of experiment.

For the purpose of verifying the actual performance of the proposed algorithm, we conducted an experiment in our laboratory. Due to the high accuracy, we applied UWB to collect the information of distance. Figure 11 shows we deployed eight anchor nodes, and the pedestrian who held the UWB in hand moved uniformly through the trajectory from the red mark point to the blue mark point; the green mark point was the anchor node, and the black mark point was the sample point. The coordinates of the beacon nodes were (0.4 m 4.1 m), (3.3 m 0.6 m), (3.6 m 3.0 m), (4.2 m 1.2 m), (5.4 m 4.2 m), (9.1 m 1.2 m), (9.6 m 3.6 m), and (11.40 m 5.18 m), respectively. The initial state vector was $\hat{x}=\left[\begin{array}{cccc} 1.8\;m & 6.0\;m & 0\;m/s & 0.6/s \end{array}\right]$. In order to avoid the receiver receiving the UWB signal reflected from the ground, the pedestrian took the mobile node in hand, which was 1.2 m above the ground. The laboratory was 12.6 m long and 6.6 m wide. Because there were obstacles such as persons, desks, and so on in the room, the measured values interfered with NLOS factors. In addition, when someone was walking in the room during the measurement, it aggravated the interference of NLOS factors. Each time the mobile node moved 0.6 m, a sampling was performed at the black point in Figure 11 and was done 30 times for each anchor node. Each anchor node collected 20 measurements at each sample interval with the average value as the final measurements handled by localization algorithm.

**Figure 12:**
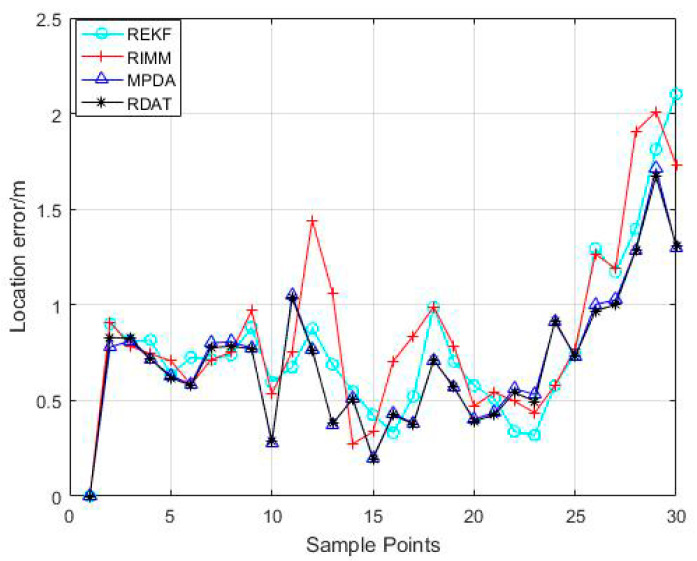
The localization error of sample points.

**Figure 13:**
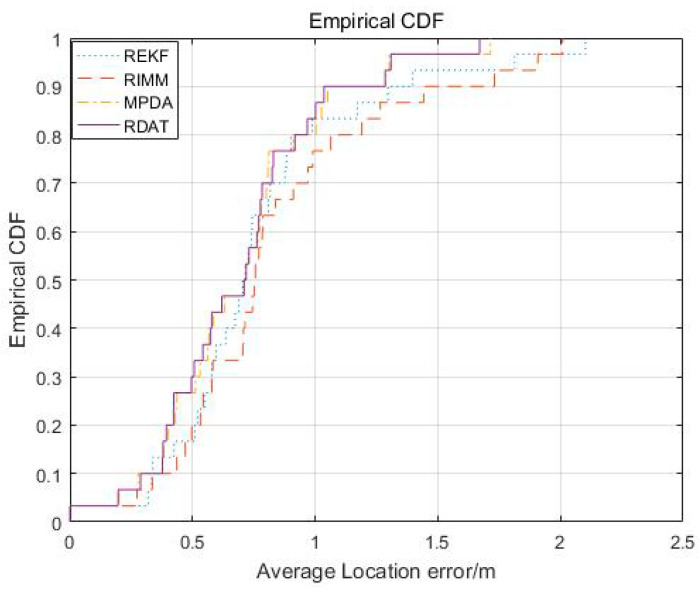
The CDF result of localization error.

The localization error distribution of each sample point is shown in Figure 12; the x-axis represents the order of the sample point, and the y-axis represents the positioning error at the corresponding sampling point in meters. The CDF is shown in Figure 13. From Figure 12, the positioning performance of the RDAT was better than REKF, RIMM, and MPDA in most sample points, and the corresponding positioning error was less than one meter. From Figure 13, the 90% error of the RDAT was less than 1.036 m, and the CDF tended to one at a localization error of less than 1.67 m, while the 90% errors of REKF, RIMM, and MPDA were achieved at 1.297 m, 1.442 m, and 1.051 m, respectively. Through the CDF, the location error of RDAT was less than 1.67 m in the whole track. Although the performance of RDAT in a real experiment was not as outstanding as in simulation, it was just because there were too many types of noise in the real environment; the algorithm we proposed still has excellent performance in most cases. Thus, there is improvement to be made on this question in the future.

## 6. Conclusions

This paper proposes am NLOS identification and suppression indoor localization algorithm. The anchor nodes participating in the positioning were divided into several subgroups, and the position estimates of the target nodes obtained by each subgroup were checked through hypothesis testing. The position estimates polluted by NLOS errors were discarded, thereby improving subsequent positioning accuracy. For the position estimation that passed the hypothesis test, the clustering probability matrix was used to improve the method of calculating the association probability to get the final target position estimation. For the case where no position estimation passed the hypothesis testing continuously, the NLOS tracking method was used to obtain the position estimation, which increased the robustness of the algorithm. Simulation results verify that the performance of the proposed algorithm was better than REKF, RIMM, and MPDA, and RDAT had high positioning accuracy when the NLOS error probability was relatively small. When the NLOS error probability was large, the positioning accuracy was reduced, thus there is improvement to be achieved in the future.

## Figures and Tables

**Table 1:** Gaussian distribution parameter.

Parameter	Sign	Values
The number of anchor nodes	M	6
NLOS error probability	$P_{NLOS}$	0.5
The measurement noise	$N\left(0,\sigma_{G}^{2}\right)$	$N\left(0,1^{2}\right)$
The NLOS errors	$N\left(\mu_{NLOS},\sigma_{NLOS}^{2}\right)$	$N\left(5,6^{2}\right)$

NLOS: non-line-of-sight.

**Table 2:** Uniform distribution parameter.

Parameter	Sign	Values
The number of anchor nodes	M	6
NLOS error probability	$P_{NLOS}$	0.5
The measurement noise	$N\left(0,\sigma_{G}^{2}\right)$	$N\left(0,1^{2}\right)$
The NLOS errors	U(a,b)	U(0,14)

**Table 3:** Index distribution parameter.

Parameter	Sign	Values
The number of anchor nodes	M	6
NLOS error probability	$P_{NLOS}$	0.5
The measurement noise	$N\left(0,\sigma_{G}^{2}\right)$	$N\left(0,1^{2}\right)$
The NLOS errors	E(λ)	E(8)
